# Predicting the global distribution of *Reaumuria songarica* under climate change based on optimized MaxEnt modeling

**DOI:** 10.3389/fpls.2026.1798185

**Published:** 2026-05-12

**Authors:** Yiming He, Liping Zhou, Ke Lu, Mili Liu, Guodong Zhu, Yizhong Duan

**Affiliations:** 1 College of Advanced Agricultural Sciences, Yulin University, Yulin, China; 2 Yulin Research Institute of Genuine Herbs of Qin Medicine Yulin University, Yulin, China; 3 Key Laboratory of Resource Biology and Biotechnology in Western China, Ministry of Education, College of Life Sciences, Northwest University, Xi’an, China

**Keywords:** climate change, environmental variables, MaxEnt, potential suitable distribution, *Reaumuria songarica*

## Abstract

**Introduction:**

*Reaumuria songarica* (Tamarixaceae) is a small shrub characterized by its strong resistance to drought, saline-alkali conditions, and wind erosion. To establish a theoretical foundation for its effective protection and utilization, this study investigated the global distribution dynamics of the species under current and future climate scenarios.

**Methods:**

Global distribution data for *R. songarica*, encompassing 278 records, alongside information on 30 environmental and climatic factors were compiled. The Maximum Entropy (MaxEnt) model was employed to simulate the globally suitable distribution areas for the species.

**Results:**

The optimized MaxEnt model demonstrates robust predictive performance (AUC = 0.963, TSS = 0.877). Key variables influencing the distribution of *R. songarica* include Ultraviolet-B radiation seasonality (UVB-2), mean temperature of the coldest quarter (Bio11), and annual precipitation (Bio12), contributing 37.8%, 30.2%, and 24.9%, respectively. Currently, the total suitable area for *R. songarica* spans 46.44 × 10^6^ km², with the core suitable zone concentrated in the temperate arid and semi-arid regions of the Eurasian continent. Under future scenarios (SSP126, SSP245, SSP370, SSP585), the potential suitable distribution areas for *R. songarica* exhibit a continuous reduction trend without any signs of expansion. The rate of reduction significantly increases with higher emission intensities, particularly under the high-emission scenarios of SSP370 and SSP585. The areas of contraction are primarily concentrated in central North America, the periphery of the core region in Central Asia, and the western edges of Eurasia. Center-of-mass migration results indicate that the future core suitable area for *R. songarica* will shift toward the Central Asia-Xinjiang-Qilian Mountains line in the central-eastern and eastern segments.

**Discussion:**

This study provides a theoretical foundation for delineating habitat protection areas, facilitating population restoration, managing resources, and implementing regional desert ecological management for *R. songarica*.

## 1 Introduction

Climate serves as the primary abiotic factor influencing species distribution, function, and change within a given region (Dormann, 2007). A combination of distinct climatic factors collectively delineates the complex geographical boundaries of habitats and distributions (Cetin et al., 2023). These varied climatic elements exert direct and indirect influences on species distribution ranges and scales, thereby significantly affecting shifts in biological distribution areas under climate change scenarios. Warming climates are inducing profound and extensive alterations in the geographic distribution of organisms (Xiong et al., 2023). According to the “Fourth National Climate Change Assessment Report,” data indicate that China’s warming accelerated to 0.27 °C per decade from 1960 to 2019, accompanied by an increasing trend in precipitation. These climatic changes, in conjunction with the biological habits of *R. songarica*, will substantially modify its spatial geographic range (Thuiller, 2007). Many scholars contend that thermal conditions significantly influence plant distribution in arid regions (An et al., 2023). In light of global climate change, the survival of plants in these areas is under considerable threat (Lu et al., 2020). Analyzing the effects of climate change on geographic distribution patterns of plant species can establish a foundation for conservation efforts in arid environments (Shi et al., 2023). Consequently, exploring shifts in species potential distribution within the context of impending climate change not only enhances natural resource management but also enriches our understanding of the interplay between biodiversity and ecosystems. This approach offers a robust information base for formulating more precise and effective conservation and management strategies.

Species distribution models (SDMs) establish relationships between species distribution patterns and corresponding environmental variables by correlating species distribution sample data with information on environmental variables. These models subsequently apply these relationships to study areas to estimate the distributions of target species (Xu et al., 2015). SDMs are extensively utilized for predicting species distributions in the context of climate change (He et al., 2023), forecasting the expansion zones of invasive species (Giulian et al., 2024), and assessing the impacts of regional climate change on plant communities and ecological functions (Zhang et al., 2011). Throughout the model development process, variations in algorithms and differing approaches to selecting and processing environmental variables and data sources have resulted in the emergence of multiple distinct models. These include the Maximum Entropy Model (MaxEnt), Artificial Neural Network Model (ANN), Generalized Linear Model (GLM), Decision Tree Model (DTM), and Generalized Additive Model (GAM). Among the various species distribution models (SDMs) available, the Maximum Entropy (MaxEnt) model is widely utilized due to its robust algorithm and precise predictive capabilities (Syfert et al., 2013). MaxEnt theory conceptualizes species-habitat relationships as a cohesive system, identifying stable species-environment interactions by calculating state parameters at maximum entropy. This framework effectively estimates species distribution patterns (Tang et al., 2021). Often integrated with ArcGIS, the MaxEnt model is essential for predicting habitat shifts for endangered species. For instance, it has been employed to forecast the potential distribution areas and priority conservation zones for *Nitraria* L. and *Epimedium brevicornu* (Lu et al., 2024; Liu et al., 2025).

*Reaumuria songarica* (Tamarixaceae) is a small shrub known for its strong resistance to drought, saline-alkali conditions, and wind-sand exposure (Lin et al., 1985; Wang et al., 2024). Its extensive root system penetrates deeply into the soil, allowing it to absorb water and nutrients while performing essential ecological functions such as soil and water conservation, windbreak, and sand fixation (Hu et al., 2024). Additionally, it provides habitats for numerous wildlife species and constitutes a vital component of Central Asian desert ecosystems (Shi et al., 2013). This species forms the most widespread strip-like community type and plays a crucial role in maintaining and restoring fragile desert ecosystems in Northwest China (Zhang Y. et al., 2023). Current research on *R. songarica* primarily addresses its taxonomy, salt stress tolerance, functional traits, molecular biology characteristics, and seed germination (Li et al., 2015; Fan et al., 2020; Shen et al., 2023; Shi et al., 2020; Song et al., 2024). While Wang et al. (2024) predicted its distribution within China’s northern deserts using 184 occurrence points, a global-scale analysis with a larger dataset is necessary to fully understand its migration routes and accurately delineate protected areas.

In this study, we focus on clarifying the response patterns of *R. songarica* to climate change. The specific objectives are as follows: (1) to identify and quantify the key climatic factors that determine its current distribution; (2) to delineate the current and future climatically suitable habitats of *R. songarica* and grade their suitability levels; and (3) to reveal the spatiotemporal dynamics, centroid shifts, and evolutionary trends of its suitable habitats under future climate change scenarios. This study improves our understanding of the adaptive strategies and survival pressures facing *R. songarica* under climate change, reveals the distribution patterns of its natural populations in response to future climate change, and provides an important theoretical and scientific basis for the conservation of its wild resources, as well as the formulation of targeted management and sustainable utilization strategies.

## 2 Materials and methods

### 2.1 Data collection

**Figure 1:**
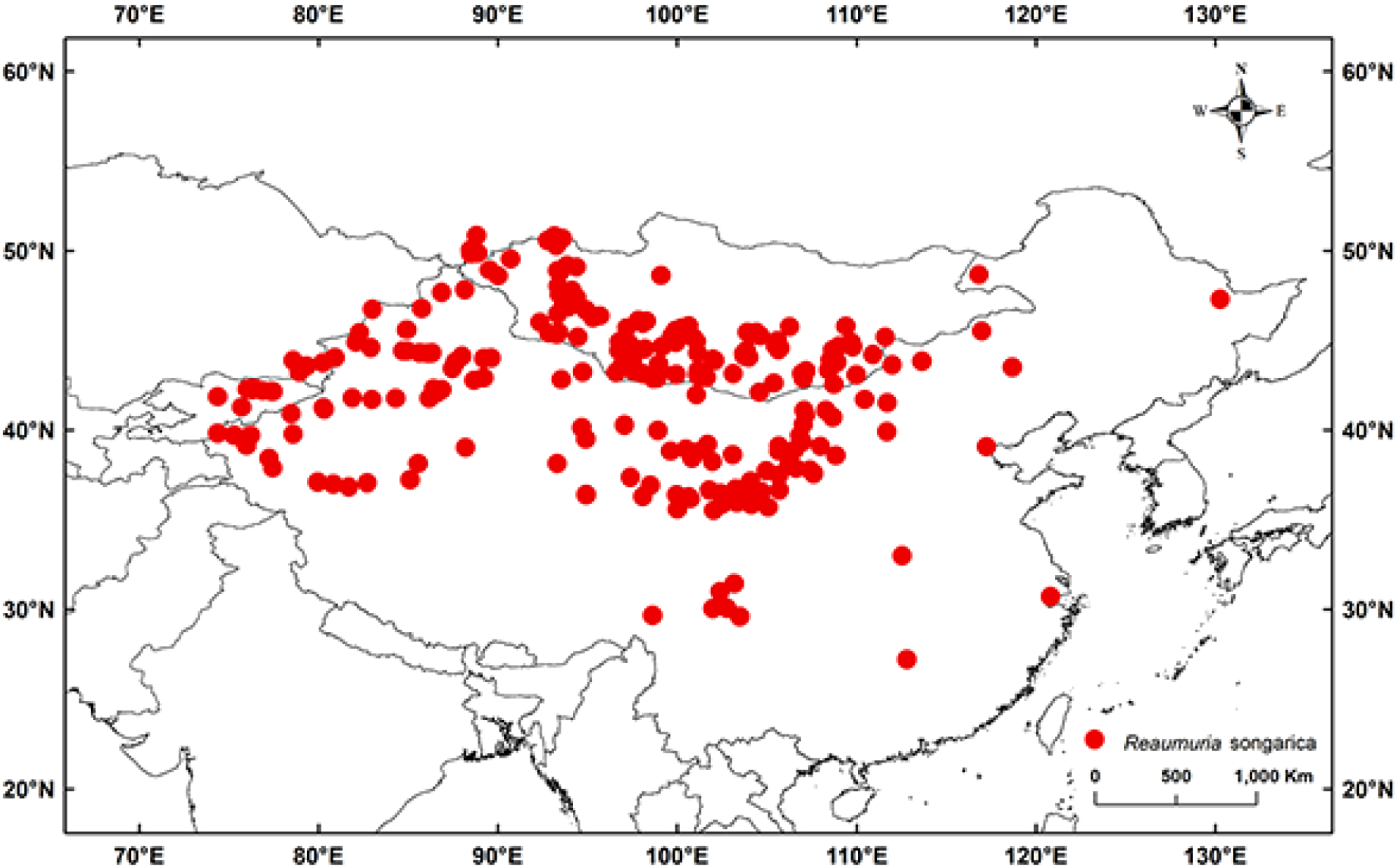
Locations of distribution points of *R. songarica* across the world.

We examined the distribution data of *R. songarica* from the Global Biodiversity Information Network database (GBIF, http://www.gbif.org). The global distribution data of the *R. songarica* was processed using ArcGIS 10.4. Then, the “CoordinateCleaner” package in R 3.6.3 was used to clean the duplicate data, in order to eliminate the data distributed in water bodies, cities and surrounding areas. At the same time, we cross-referenced all occurrence records with botanical atlases and available literature, manually removing those with inaccurate geographic locations. Following this, we utilized ENMTools for spatial deduplication to remove duplicate records, mislocated data, and information outside the study area. A spatial rasterization process with a resolution of 5 × 5 km was implemented to ensure that each geographic unit retained only the most representative distribution points (Yong et al., 2025). Ultimately, we obtained 278 valid distribution points that met the research criteria, thereby creating a reliable dataset for spatial analysis (**Figure 1**).

### 2.2 Predictor variables

This study employed four soil variables, four global UVB radiation variables (UVB1-4), nineteen bioclimatic variables (bio1-bio19), and three topographic factors (DEM/Aspect/Slope) to develop species distribution models (**Table 1**). The four soil variables were sourced from the Harmonized World Soil Database (HWSD, https://www.fao.org/soils-portal/en/), while the four global UVB radiation variables were obtained from the gIUV database, a global UV-B radiation dataset for macroecological studies (http://www.ufz.de/gluv/). Additionally, the nineteen bioclimatic variables and three topographic factors were derived from WorldClim2.1 (http://www.worldclim.org/).

**Table 1:** Environmental variables and their contributions and suitable value ranges.

Environment variable	Description	Unit	Contribution (%)	Importance (%)	Total suitable range
Bio1	Annual Mean Temperature	°C	×	×	×
Bio2	Mean diurnal temperature range	°C	×	×	×
Bio3	Isothermality (Bio2/Bio7) (×100)	-	×	×	×
Bio4	Seasonal variation coefficient of temperature (standard deviation×100)	-	3.6	2.3	1033.04-1534.37
Bio5	Max Temperature of Warmest Month		×	×	×
Bio6	Min Temperature of Coldest Month	°C	×	×	×
Bio7	Temperature Annual Range	°C	×	×	×
Bio8	Mean temperature of the wettest quarter	°C	×	×	×
Bio9	Mean Temperature of Driest Quarter	°C	×	×	×
Bio10	Mean Temperature of Warmest Quarter	°C	×	×	×
Bio11	Mean Temperature of Coldest Quarter	°C	30.2	40.4	−15.91-−3.62
Bio12	Annual Precipitation	mm	24.9	6.6	44.46-164.49
Bio13	Precipitation of Wettest Month	mm	×	×	×
Bio14	Precipitation of Driest Month	mm	×	×	×
Bio15	Precipitation Seasonality	mm	×	×	×
Bio16	Precipitation of Wettest Quarter	mm	×	×	×
Bio17	Precipitation of the Driest Quarter	mm	×	×	×
Bio18	Precipitation of Warmest Quarter	mm	×	×	×
Bio19	Precipitation of Coldest Quarter	mm	×	×	×
S_OC	Soil Organic Carbon	g/kg	0.4	0.4	0.14-0.51
S_Sand	Soil Sand	%	1.6	1.8	26.70-34.06 45.22-57.34
S_BD	Soil Bulk density	g/cm^3^	1.0	4.6	1.36-1.49
S_T	Soil Texture	-	0.5	0.5	1.82-2.65
UVB1	Annual Mean UV-B	J/m^2^/day	×	×	×
UVB2	UV-B Seasonality	J/m^2^/day	37.8	43.8	165841.44-199758.31
UVB3	Mean UV-B of lightest Month	J/m^2^/day	×	×	×
UVB4	Mean UV-B of Lowest Month	J/m^2^/day	×	×	×
DEM	Digital Elevation Model	m	×	×	×
Aspect	Aspect	-	×	×	×
Slope	Slope	°	×	×	×

The variables isothermality (Bio3), temperature seasonality (Bio4), Soil texture (ST) and Aspect are expressed as dimensionless indices or percentages. Given their nature as ratios or standardized scales, they are presented. without physical units in this table. Note: Variables without any values (indicated by ×) were removed because of high cross-correlations.

The currently available climate data covers the period from the 1970s to the 2000s. Future climate projections are based on the Sixth Coupled Model Intercomparison Project (CMIP6) data, which includes four socio-economic pathways: SSP126, SSP245, SSP370 and SSP585. These pathways correspond to the lowest, moderate, high, and maximum greenhouse gas emission scenarios, respectively. The future climate data are derived from four distinct time periods: 2021-2040 (2030s), 2041-2060 (2050s), 2061-2080 (2070s), and 2081-2100 (2090s). The future projections utilize the second-generation National Climate Center’s Medium-Resolution Climate System Model (BCC-CSM2-MR).

### 2.3 Bioclimatic variables screening

An abundance of environmental factors can result in model overfitting problems like multicollinearity and autocorrelation among these factors (Gan et al., 2025). Initially, pairwise correlation assessments were performed on all environmental variables, which were subsequently integrated into the following MaxEnt pre-experimental model. Environmental variables with correlation coefficients |r| < 0.8 were eliminated. For cases where |r| ≥ 0.8, variables showing substantial contributions in the initial experiments were preserved. In the end, eight environmentally and statistically significant data points were chosen for modeling (**Table 1**).

### 2.4 MaxEnt model establishment, optimization and evaluation

Species distribution models were constructed using the MaxEnt algorithm. However, as a complex machine learning model, MaxEnt is sensitive to sampling bias, and its complexity is significantly affected by two key parameters: feature combinations (FC) and the regularization multiplier (RM). The FC set included linear (L), quadratic (Q), product (P), threshold (T), and hinge (H) features. To minimize the impact of overfitting and enhance model transferability, we employed the ENMeval package in R 3.6.3 to evaluate multiple parameter combinations. We tested cross-combinations of FC (LQPTH) and RM values ranging from 0 to 4 at 0.1 increments, and the optimal parameter combination was selected based on the lowest delta AICc (Daniela and Derya, 2024). Upon importing the distribution data of *R. songarica* and the necessary environmental factors into the MaxEnt (V3.4.4) model, the parameters were configured as follows: The dataset underwent random sampling, with 75% assigned to Training Data and 25% to Testing Data. The number of replicates was designated as 10, utilizing the “Bootstrap” iteration method (Zhao et al., 2025). The maximum background point count was established at 10,000, with the remaining parameters left at their default settings. The output file format was specified as “Cloglog”, and the file type was set as “asc”.

The Jackknife method was utilized to evaluate the impact of environmental factors, while the model’s accuracy was assessed through the receiver operating characteristic (ROC) curve (Yao et al., 2025). The model’s performance was quantified by the area under the curve (AUC), which ranges between 0.5 and 1, with higher values indicating greater predictive reliability (Herrando-Moraira et al., 2020). AUC values closer to 1.0 suggest a more reliable model, with values exceeding 0.9 denoting outstanding predictive capability (Ao et al., 2025). Despite its significance, AUC alone may not suffice as a predictive indicator for spatial distribution modeling (Li et al., 2023). Hence, we introduce supplementary metrics such as True Skill Statistics (TSS) and the Kappa consistency test. By selecting maxSSS (maximum sum of sensitivity and specificity) as the threshold, we computed the average TSS from 10 repetitions of the MaxEnt model to assess its performance (Liu et al., 2015).

### 2.5 The classification of suitable areas of *R. songarica*

The ensemble models’ continuous habitat suitability outputs were rescaled to a range of 0–1 and then classified into five categories based on the Jenks natural breaks method: Unsuitable (0.0-0.2), Poorly suitable (0.2-0.4), Moderately suitable (0.4-0.6), Highly suitable (0.6-0.8), and Extremely highly suitable (0.8-1.0). The total area of each suitability class was quantified for every time slice and scenario. To evaluate changes in range, the continuous suitability maps were converted into binary layers by applying a minimum suitability threshold of 0.2, which corresponds to the minimum value of the low-suitability class. By overlaying binary maps of present and future conditions, alterations in range size, expansions, contractions, and areas remaining stable under varying climates were determined. Spatial analyses were conducted utilizing SDMtoolbox v2.6 within ArcGIS (Li et al., 2024).

### 2.6 Area change and transfer of suitable habitat distribution center

A systematic quantitative assessment of potential distribution areas for *R. songarica* was conducted using spatial analysis techniques from the SDMTools module. Initially, the “Quick Reclassify to Binary” tool was utilized to convert the Habitat Suitability Index (HSI) output from the MaxEnt model into binary form. The Maximum test sensitivity plus specificity logistic threshold was applied as the classification criterion. This process categorized areas with HSI values exceeding the threshold as potential suitable zones, while those below the threshold were designated as unsuitable zones, resulting in the creation of a spatial distribution binary raster dataset.

Using the “Distribution Change Between Binary Species Distribution Models (SDMs)” tool, we performed spatial overlay analysis on binary distribution maps from multiple time periods. The types of distribution changes were classified into four categories: 0 for consistently unsuitable areas, 1 for deteriorating suitable areas (areas with decreasing distribution), 2 for consistently suitable areas (areas with stable distribution), and 3 for newly emerging suitable areas (areas with expanding distribution). This method systematically measures the dynamic changes in suitable areas over different time frames (Gufi et al., 2023). To further examine the spatial development of the suitable distribution zone for *R. songarica*, we applied the spatial centroid calculation technique from SDMTools. By determining the geometric center coordinates of binary distribution grids from various time periods, we established a trajectory of centroid migration. This approach accurately traces the spatial shifts and evolutionary paths of the species’ potential distribution range by calculating the weighted center points of suitable area grids for each period, offering quantitative insights into the alterations in distribution patterns (Shen et al., 2025).

### 2.7 Protection status and key protected areas of *R. songarica*

Access to global nature reserve distribution data was obtained from the world’s reserve data center (https://www.protectedplanet.net/en/thematic-areas/wdpa?tab=WDPA). ArcGIS 10.4 was employed to analyze changes in the extent of protected areas and to identify potential suitable habitats for *R. songarica* across different periods within these protected regions.

## 3 Results

### 3.1 MaxEnt model optimization and accuracy evaluation results

**Figure 2:**
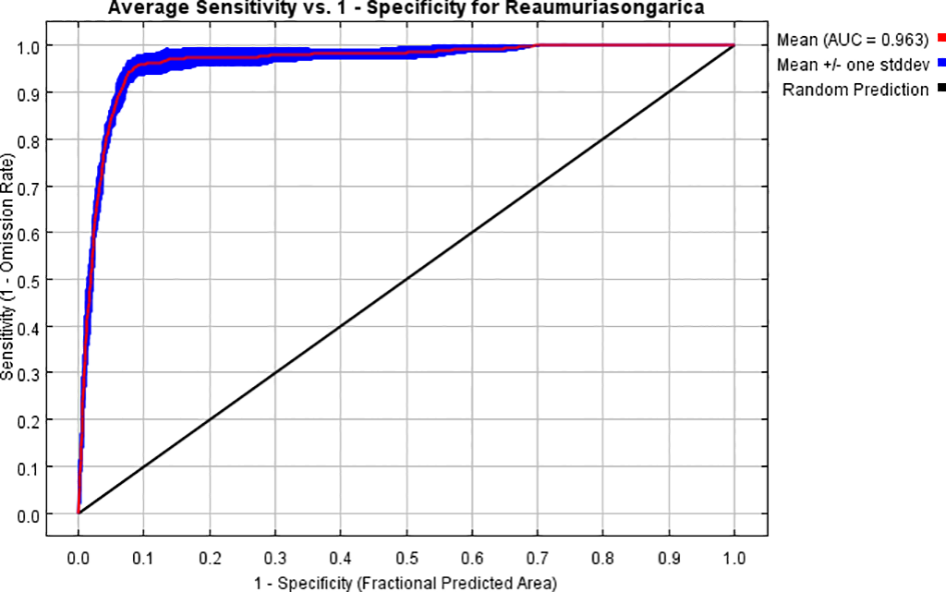
ROC curve for *R. songarica* using the optimized MaxEnt model.

**Figure 3:**
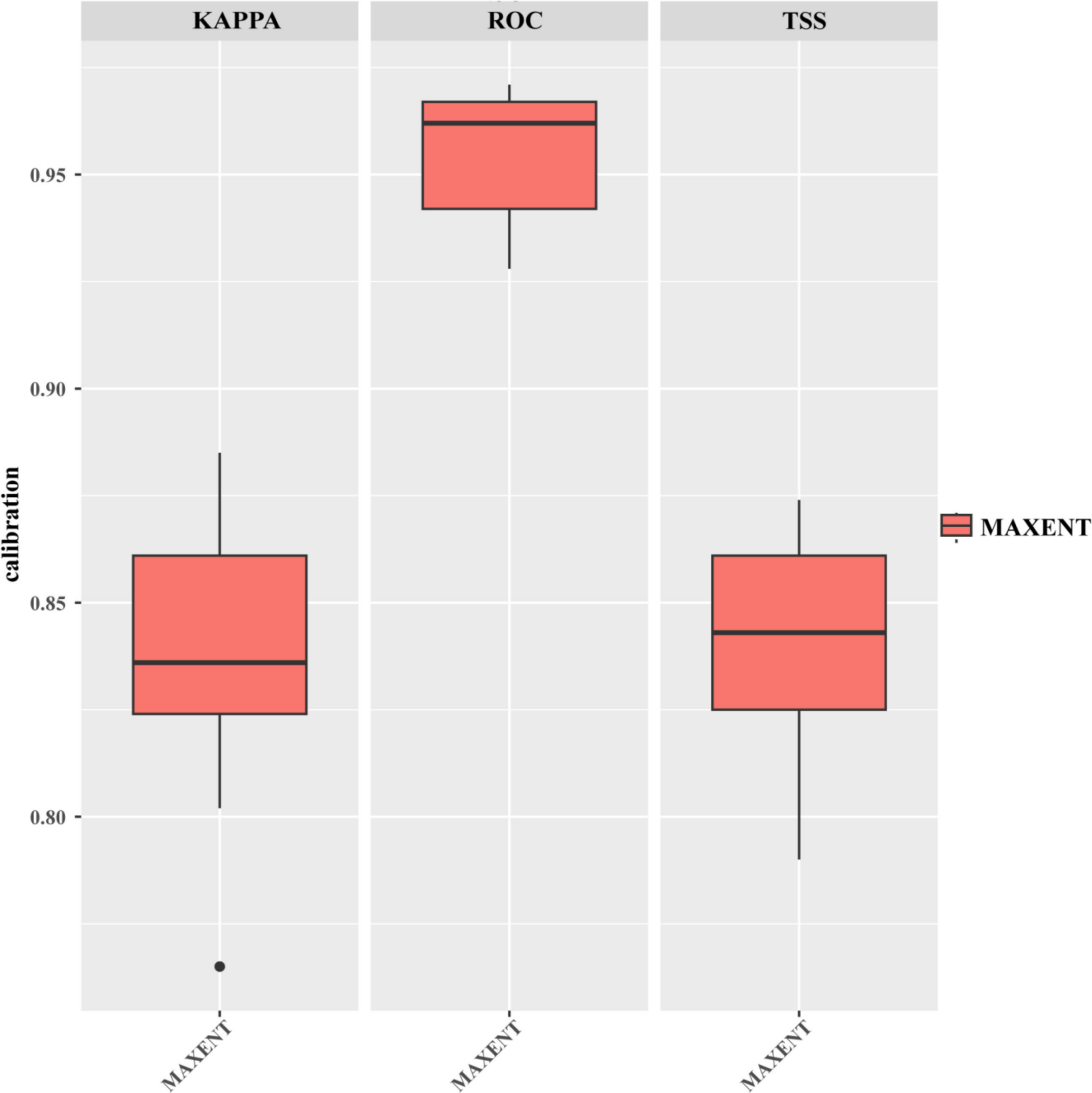
KAPPA-ROC-TSS for *R. songarica* using the optimized MaxEnt model.

As shown in **Table 2**, the combination of FC = LQHP and RM = 0.5 yielded the best performance (delta AICc = 0), whereas the default parameters (FC = LQHPT, RM = 1) resulted in a substantially higher delta AICc of 36.1071. Furthermore, compared to the default setting, the optimized model exhibited marked improvements, with decreases of approximately 81.68% in AUC.DIFF and 52.90% in OR10. The MaxEnt model’s predictions for *R. songarica* distribution under current climatic conditions yielded an Area Under the Curve (AUC) of 0.963. The Receiver Operating Characteristic (ROC) curve displayed a distinct upward-left shift, indicating exceptionally high prediction accuracy, meeting the criteria for outstanding predictive capability (**Figure 2**). Both the Cohen’s Kappa (KAPPA) and the True Skill Statistic (TSS) values exceed 0.80, further affirming the model’s elevated predictive sensitivity (**Figure 3**). This model enables precise forecasting of *R. songarica* distribution across globally suitable regions.

**Table 2:** Evaluation metrics of MaxEnt model generated by ENMeval.

Parameter settings	FC	RM	Delta.AICc	AUC.DIFF	OR10	AUC	TSS
Default	LQHPT	1.0	36.1071	0.0513	0.2529	0.847 ± 0.013	0.798 ± 0.021
Optimized	LQHP	0.5	0	0.0094	0.1191	0.963 ± 0.009	0.877 ± 0.025

FC, feature combination; RM regularization multiplier.

### 3.2 Important environmental variables preference

**Figure 4:**
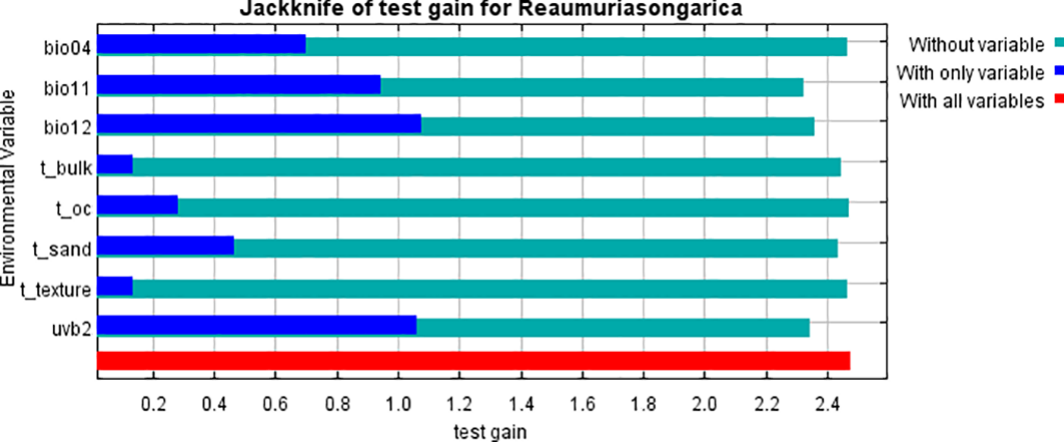
The effect of environmental variables on the distribution of *R. songarica* was evaluated by the knife-cutting method.

**Figure 5:**
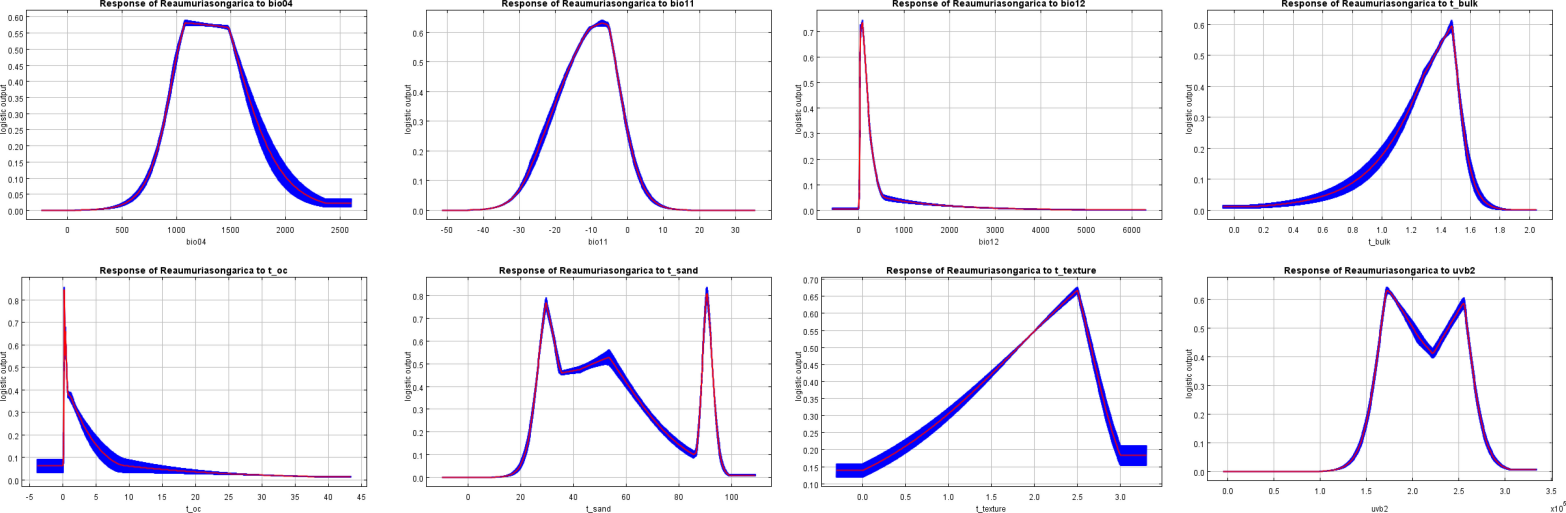
Response curves for key environmental predictors in the species distribution model for *R. songarica* (The red line represents the average value of all candidate models, and the blue ranges, indicates the standard deviation, the same below).

This study utilized outputs from the MaxEnt model, along with the regularized training gain contribution rate, test gain rate, replacement contribution rate, and one-way response curves derived from the knife-cut method, to identify the dominant environmental factors influencing the geographic distribution of *R. songarica* (**Figures 4**, **5**; **Table 1**). Three factors emerged as primary determinants of *R. songarica* distribution: UV-B seasonality (UVB-2), mean temperature of the coldest quarter (Bio11), and annual precipitation (Bio12), with contribution rates of 37.8%, 30.2%, and 24.9%, respectively. UVB-2 and Bio11 also exhibited the highest replacement importance values, at 43.8% and 40.4%, indicating their significant influence on the predictive accuracy of the model. Other variables, including temperature seasonality (Bio4) and four soil factors (S_Sand, S_BD, S_T, S_OC), demonstrated low contribution rates ranging from 0.4% to 1.6%, suggesting secondary roles. The optimal ranges for these key factors, defined by an occurrence probability greater than 0.5 (Ren et al., 2025), are summarized as follows (**Figure 5**; **Table 1**): UVB-2: 165, 841.44-199, 758.31 J·m^-^²·day^-^¹; Bio11: −15.91 - −3.62 °C; Bio12: 44.46-164.49 mm; Bio4: 1, 033.04-1, 534.37; and soil variables: ranges as detailed in **Table 1**. These findings indicate that radiation, low-temperature stress during winter, and water availability serve as the primary environmental filters influencing the distribution of *R. songarica*.

### 3.3 Potential distribution areas under current climate

**Figure 6:**
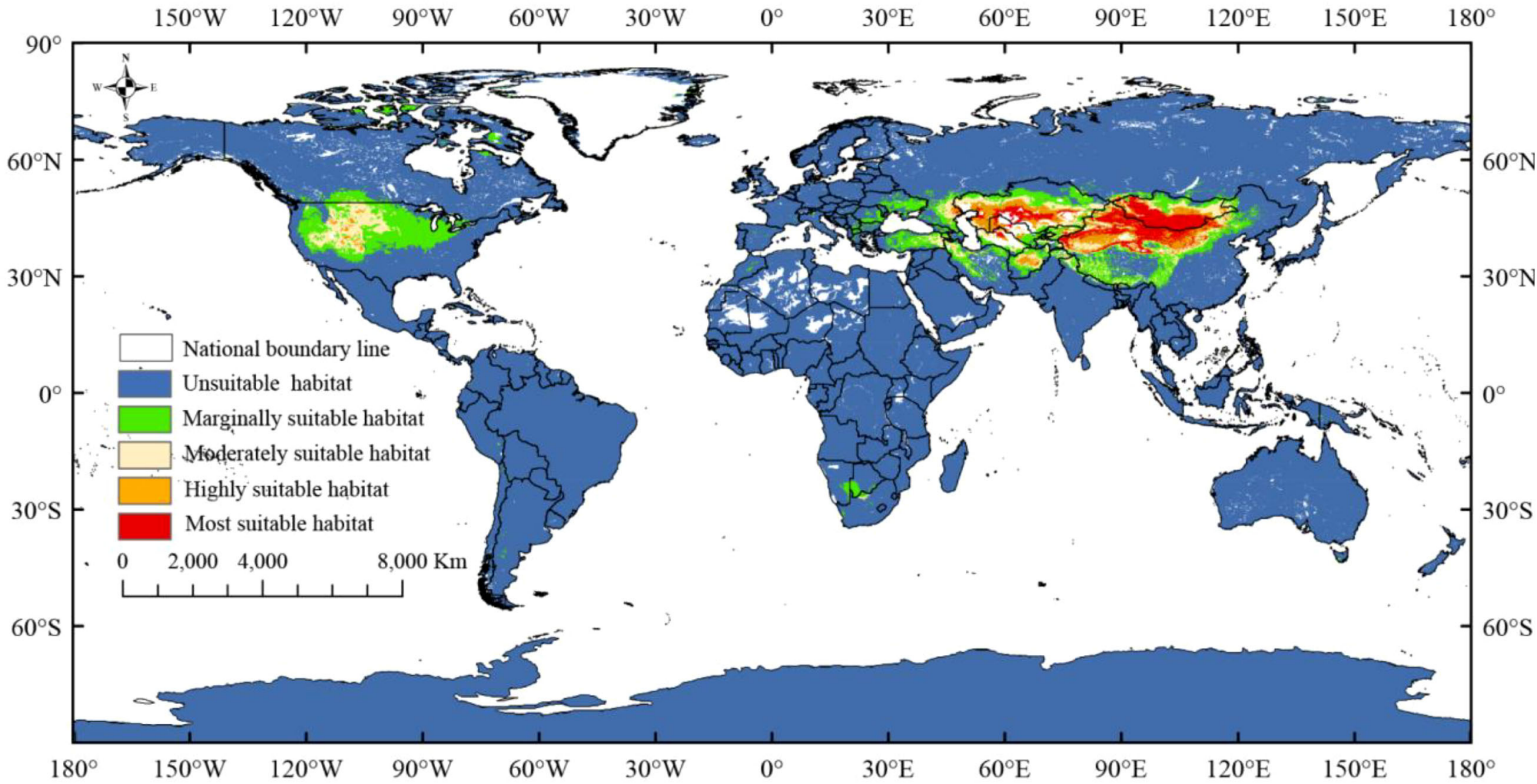
Maps of current potential habitat of *R. songarica* across the world.

The total current suitable habitat for *R. songarica* spans 46.44 × 10^6^ km², showing a gradual decrease in suitability from the core to the periphery (**Figure 6**; **Table 3**). The highly suitable areas, encompassing 28.75% (13.35 × 10^6^ km²) and 16.13% (7.49 × 10^6^ km²) of the total, are mainly concentrated in the temperate arid and semi-arid regions of Central Asia, such as the Mongolian Plateau, Xinjiang (China), and West Asia. Moderately suitable areas cover 20.28% (9.42 × 10^6^ km²), primarily found in the central United States, while poorly suitable areas constitute the largest portion (34.82%, 16.17 × 10^6^ km²), scattered across North America, southern Africa, West Asia, and eastern China. This distribution pattern confirms that *R. songarica* is predominantly a species of Eurasian steppes and deserts, constrained by temperate continental climatic conditions.

**Table 3:** Portions of different classes of potential distribution area of *R. songarica* under current and future climate scenarios/years.

Species	Period		Poorly suitable area	Moderately suitable area	Highly suitable area	Extremely highly suitable area	Total suitable area
Area of each suitable area ×10^6^ km^2^ (change in the area compared to current)							
*R. songarica*	Current	-	16.17	9.42	7.49	13.35	46.44
	SSP126	2030	15.97 (-1.24%)	9.02 (-4.25%)	7.39 (-1.34%)	13.85 (3.75%)	46.23 (-0.45%)
		2050	15.79 (-2.35%)	7.85 (-16.67%)	7.57 (1.07%)	13.97 (4.64%)	45.18 (-2.71%)
		2070	15.49 (-4.21%)	7.75 (-17.73%)	7.49 (0.00%)	13.67 (2.40%)	44.40 (-4.39%)
		2090	15.99 (-1.11%)	7.87 (-16.45%)	7.47 (-0.27%)	13.97 (4.64%)	45.30 (-2.45%)
	SSP245	2030	15.57 (-3.71%)	8.48 (-9.98%)	7.53 (0.53%)	13.84 (3.67%)	45.42 (-2.20%)
		2050	15.89 (-1.73%)	7.75 (-17.73%)	7.44 (-0.67%)	13.97 (4.64%)	45.05 (-2.99%)
		2070	15.39 (-4.82%)	7.65 (-18.79%)	7.48 (-0.13%)	13.77 (3.15%)	44.29 (-4.63%)
		2090	13.60 (-15.89%)	8.83 (-6.26%)	6.90 (-7.88%)	13.34 (-0.07%)	42.67 (-8.12%)
	SSP370	2030	15.93 (-1.48%)	9.69 (2.87%)	7.18 (-4.14%)	13.41 (0.45%)	46.21 (-0.50%)
		2050	15.05 (-6.93%)	9.56 (1.49%)	7.00 (-6.54%)	13.09 (-1.95%)	44.70 (-3.75%)
		2070	15.44 (-4.51%)	7.31 (-22.40%)	6.38 (-14.82%)	12.92 (-3.22%)	42.05 (-9.45%)
		2090	15.90 (-1.67%)	7.19 (-23.67%)	6.30 (-15.89%)	12.42 (-6.97%)	41.81 (-9.97%)
	SSP585	2030	16.07 (-0.62%)	9.22 (-2.12%)	7.19 (-4.01%)	13.15 (-1.50%)	45.63 (-1.74%)
		2050	13.41 (-17.07%)	9.43 (0.11%)	7.21 (-3.74%)	13.74 (2.92%)	43.79 (-5.71%)
		2070	15.46 (-4.39%)	7.20 (-23.57%)	6.38 (-14.82%)	12.68 (-5.02%)	41.72 (-10.16%)
		2090	15.25 (-5.69%)	7.26 (-22.93%)	6.45 (-13.88%)	12.58 (-5.76%)	41.54 (-10.55%)

### 3.4 Changes in the suitable habitat areas of *R. songarica* in the future

**Figure 7:**
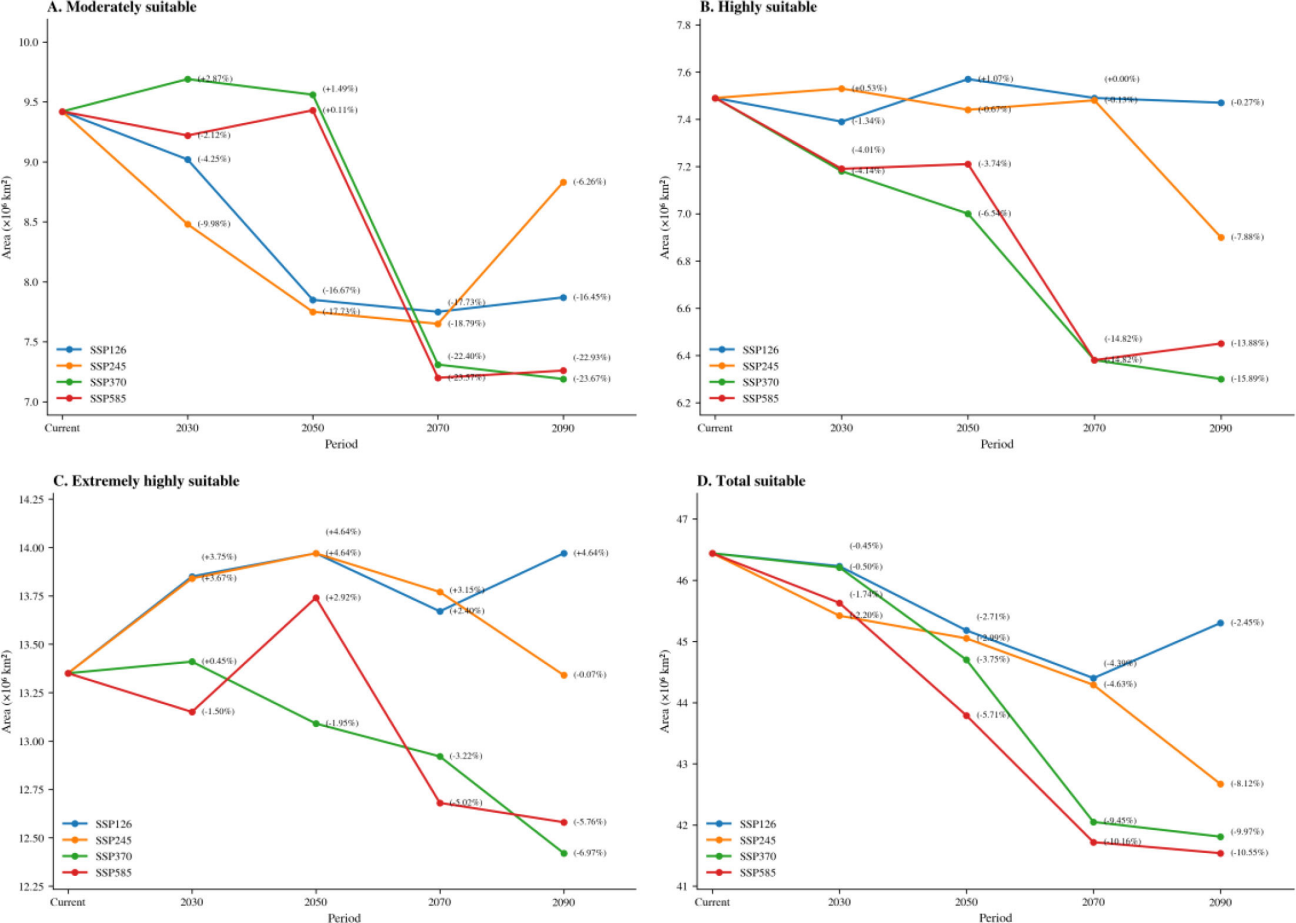
The future climate change scenarios for *R. songarica* show a greater distribution change compared to the current situation.

The potential suitable distribution areas for *R. songarica* in China under various future climate conditions indicate that, under the SSP126, SSP245, SSP370, and SSP585 emission scenarios, the current distribution area will continue to be suitable for *R. songarica*. Its primary distribution will remain concentrated in Central Asia, West Asia, and the northwestern regions of China. Nonetheless, the total suitable distribution area is expected to decline progressively over time, with the rate of contraction differing significantly among the various climate scenarios (**Figure 7**; **Table 3**).

The trends vary by scenario. Under the low-emission SSP126, the total suitable area remains relatively stable, with only a slight reduction of 0.45%-4.39% through the 2090s. Under the moderate-emission SSP245, a gradual decline is anticipated after the 2050s. By the 2090s, the total suitable area is projected to decrease by 8.12%, with contraction primarily occurring in the peripheral zones of the Central Asian core. SSP370 (High Emission): A significant decline is expected after the 2070s. Habitat loss intensifies over time, resulting in increasing fragmentation of peripheral zones. The total reduction rate reaches 9.45%–9.97% by the 2090s, affecting both the core and periphery of the Central Asian region and central North America. SSP585 (Very High Emission): This scenario forecasts the most severe impacts with the earliest onset. Following an initial gradual decline, the total suitable area contracts sharply after the 2050s. By the 2090s, the reduction rate reaches 10.16%-10.55%, leading to fragmentation of the core habitat in Central Asia and significant habitat loss in its periphery.

### 3.5 The spatial shift in potential habitats centroid in the future

**Figure 8:**
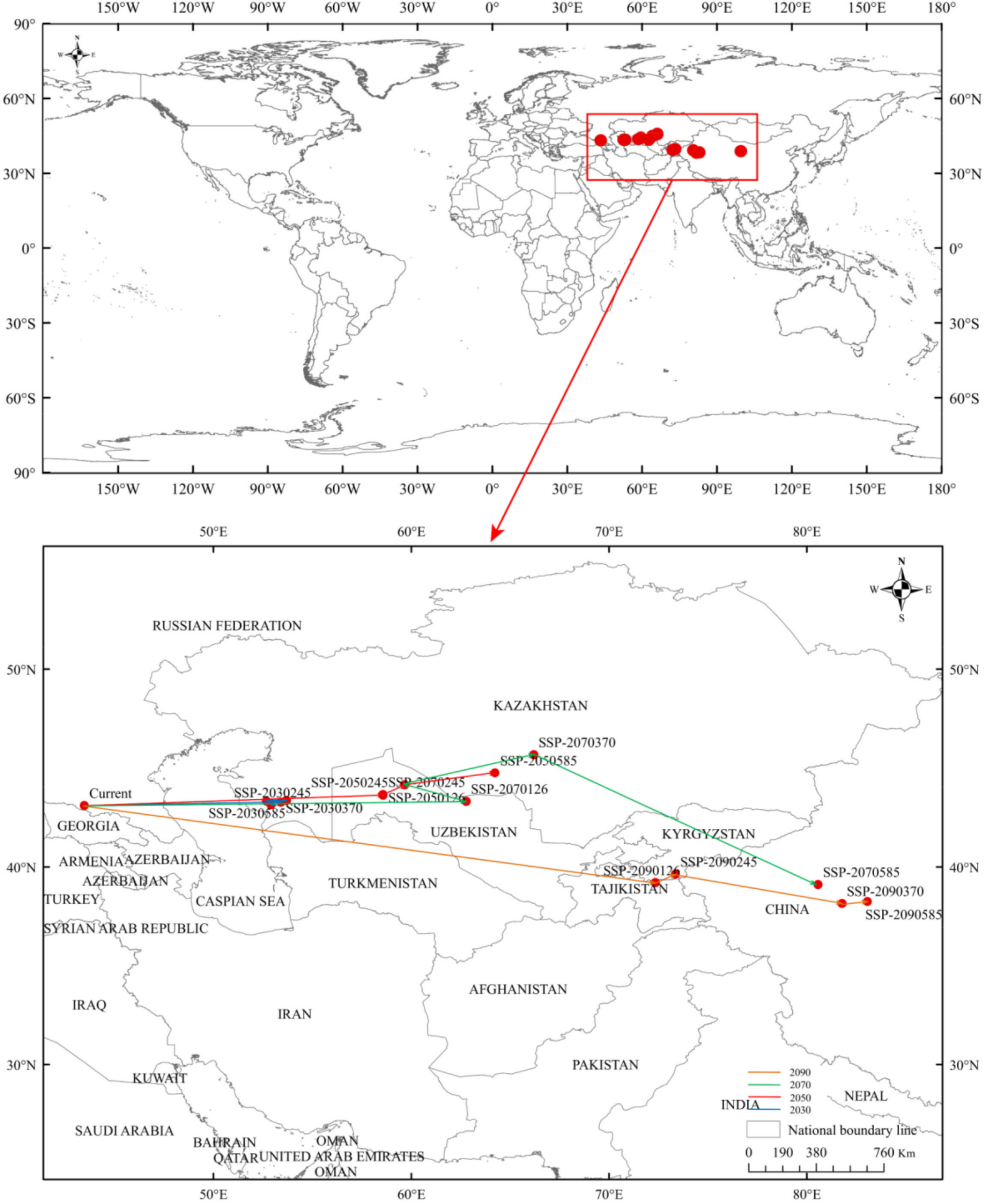
The core distributional shifts under different climate scenarios/years for *R. songarica*.

Currently, the center of suitability for *R. songarica* is situated in Russia’s North Caucasus region (approximately 43.11°N, 43.49°E), within the mountainous-arid steppe transition zone east of the Caspian Sea. As the climate warms in the future, the center of mass exhibits a distinct trend of shifting from west to east across four emission scenarios, transitioning from the Caucasus to Central Asia and subsequently toward the arid regions of Xinjiang and beyond. However, the specific pathways and intensities of migration differ among the scenarios (**Figure 8**; **Table 4**).

**Table 4:** Portions of different classes of potential distribution area of *R. songarica* under current and future climate scenarios/years.

Species	Longitude	Latitude
Current	43.48535695	43.11267372
SSP-2030126	52.69868094	43.33304547
SSP-2030585	53.69868094	43.36304547
SSP-2030370	53.41204076	43.269569
SSP-2030245	52.92206162	43.14435557
SSP-2050126	58.59550246	43.63212402
SSP-2050585	64.24936948	44.75451095
SSP-2050370	59.67621523	44.14771916
SSP-2050245	58.57550246	43.65212402
SSP-2070126	62.77978403	43.32215256
SSP-2070585	80.58571122	39.11234218
SSP-2070370	66.20160158	45.67525713
SSP-2070245	58.59550246	43.66212402
SSP-2090126	72.35032075	39.20318258
SSP-2090585	83.07382746	38.23801432
SSP-2090370	81.78633048	38.14783386
SSP-2090245	73.37252385	39.64057164

The key trends for each scenario are as follows: SSP126 (Low Emission): The center of mass exhibits a moderate eastward shift within Central Asia, with a cumulative migration distance of approximately 2, 483.7 km by the 2090s. This trajectory is marked by a series of progressive eastward movements, culminating in a significant southeastward leap into the Western Tianshan Mountains and Kyrgyzstan-Fergana Basin by the century’s end. SSP245 (Moderate Emission): The migration follows a more intricate, three-phase pattern: an initial slow eastward drift, a period of relative stability in central-western Kazakhstan during the mid-century (2070s), and a rapid southeastward leap exceeding 1, 300 km by the 2090s to the northern edge of the Fergana Basin. The total migration distance is approximately 2, 532.0 km. SSP370 (High Emission): Under this medium-to-high emission scenario, the center of mass migrates significantly into inland arid regions, covering a distance of approximately 3, 391.5 km. Following an initial eastward movement, the centroid shifts northeastward before making a considerable southeastward leap exceeding 1,500 km by the 2090s, ultimately reaching the northern edge of the Tarim Basin in Xinjiang, China. SSP585 (Very High Emission): This scenario results in the most extensive eastward migration, with a total cumulative distance of around 4, 844.5 km. The trajectory includes substantial early movements into central-western Kazakhstan, followed by a rapid southeastward advance into the Tarim Basin by the 2070s. By the 2090s, the centroid shifts further east along the northern edge of the basin, ultimately arriving at the Qilian Mountains-Hexi Corridor region in northwest China.

All four scenarios consistently indicate a spatial reorganization trend for *R. songarica*, with its suitability center gradually moving from the Caucasus-Caspian Sea region to the arid belt within Kazakhstan. Under medium-to-high emission scenarios, this shift will progress towards the eastern arid zone through the Central Asia-Xinjiang-Qilian Mountains corridor. This suggests a substantial east-west and latitudinal redistribution of the optimal climatic space for *R. songarica* within the arid belt of the Old Continent. The core suitable zone for *R. songarica* will shift gradually from the western to the central and eastern sections of the Eurasian arid belt. These findings have significant implications for regional desert ecosystem patterns, vegetation restoration, and strategies for controlling desertification.

### 3.6 Conservation status of *R. songarica*

**Figure 9:**
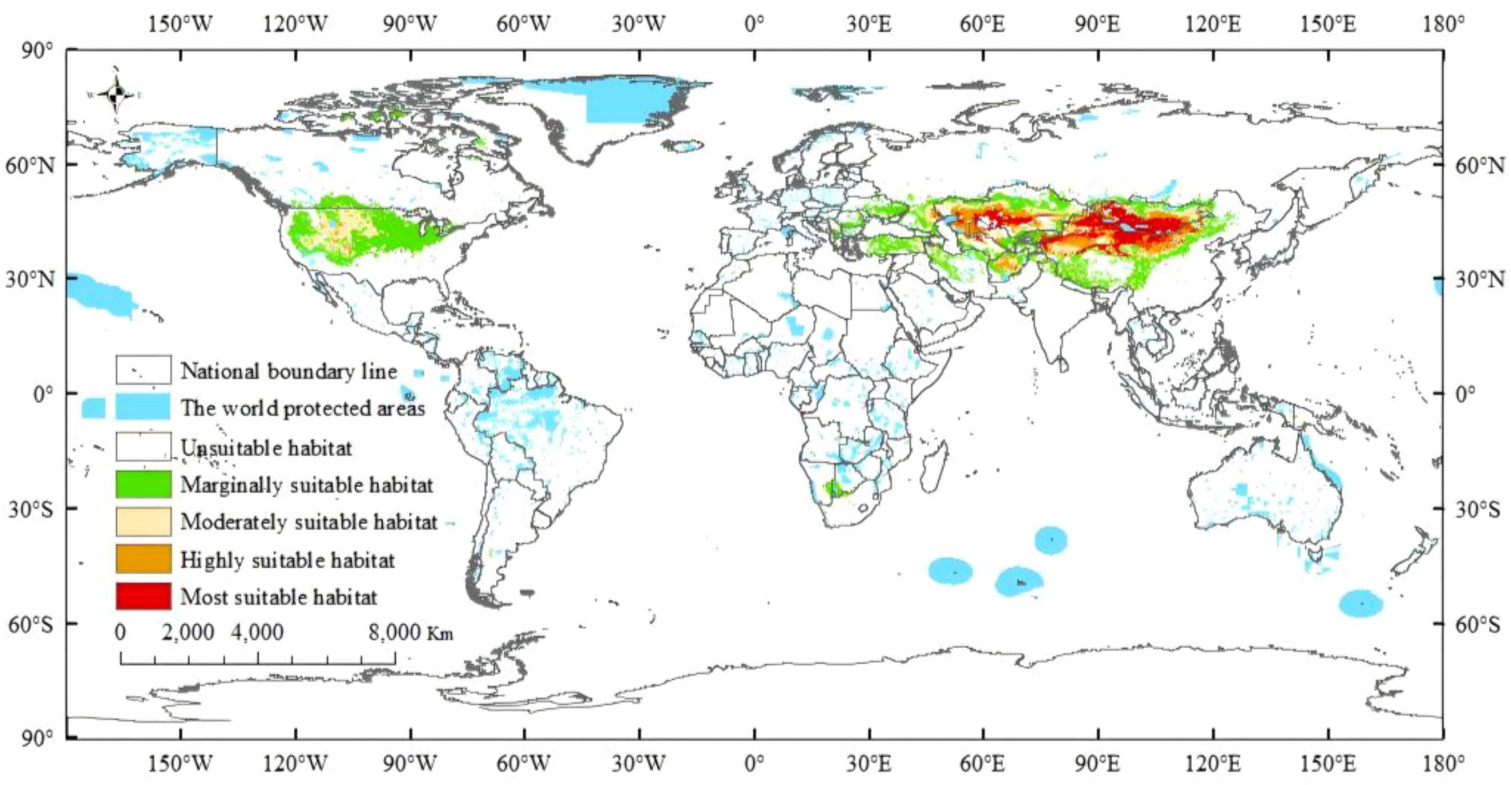
Distribution of potential suitable areas in the current protection area of *R. songarica*.

The current and future highly suitable habitats for *R. songarica* are primarily located in Central Asia, particularly in Northwest China and Southern Mongolia (**Figure 9**). However, the protected areas encompassing these habitats are relatively small and fragmented, underscoring the urgent need to enhance conservation efforts for this species in Central Asia. Under both low and high-emission climate scenarios, the extent of protected lands within highly suitable habitats exhibits minimal change despite rising greenhouse gas concentrations (**Figures 6**, **9**). In contrast, the overall potential suitable habitat for *R. songarica* appears to diminish as greenhouse gas levels increase.

## 4 Discussion

### 4.1 Model parameter optimization and reliability of prediction results

Previous scholarly research has established that the MaxEnt model serves as an ecological niche model based on probability theory and machine learning principles (Phillips and Dudík, 2008; Yang J. et al., 2025). It is characterized by a concise mathematical formulation, adaptable parameter settings, rapid convergence, high predictive accuracy, and remarkable stability. In scenarios involving small sample sizes, its predictive performance surpasses that of other ecological niche models. In this study, the MaxEnt model simulation produced an AUC value exceeding 0.8, indicating favorable predictive outcomes (**Figure 2**). Nonetheless, existing literature has demonstrated that AUC alone is insufficient for evaluating spatial distribution modeling. Consequently, we incorporated Kappa and TSS metrics, which yielded values greater than 0.80 (**Figure 3**), further validating the model’s high prediction sensitivity and its capacity to objectively represent species responses to environmental factors. Additionally, the study identified 8 environmental factors from a pool of 30 variables based on model contribution rates and permutation importance values (**Table 1** and **Figure 5**). This selection not only enhanced the spatial accuracy of the model but also improved the overall accuracy of its predictions.

### 4.2 The influence of environmental of *R. songarica*

Environmental factors, biological factors, species diffusion capabilities, and evolutionary adaptability are the primary determinants of species geographical distribution (Weldon et al., 2018; Scherrer and Guisan, 2019). However, on a regional scale, the distribution of plants is predominantly influenced by climate, with hydrothermal conditions playing a crucial role (Wang J. et al., 2023). In this study, the MaxEnt model identified UV-B Seasonality (UVB-2), Mean Temperature of Coldest Quarter (Bio11), and Annual Precipitation (Bio12) as the primary climatic factors influencing the distribution of *R. songarica*, with contribution rates of 37.8%, 30.2%, and 24.9%, respectively, yielding a cumulative contribution of 92.9%. These findings suggest that solar radiation, temperature, and precipitation collectively shape the species’ habitat, with solar radiation being the most significant factor.

Climate change driven by global warming has altered the intensity of UV-B radiation and precipitation patterns (Neale et al., 2021). Arid desert regions are particularly sensitive to variations in precipitation and light, making these factors critical in maintaining ecosystem stability (Wu et al., 2023). Ultraviolet radiation can significantly affect plant metabolism, physiology, and development (Liu et al., 2023). Wang et al. (2024) demonstrated that the seasonality of UV-B radiation profoundly influences the distribution of *R. songarica* in simulations of suitable planting areas in China, aligning with the findings of this study. Similarly, Lu et al. (2024) reported that among various environmental factors, ultraviolet radiation accounted for 65.5% of the effects on *Nitraria* L., underscoring its importance in the growth and distribution of plants in arid regions. Ultraviolet-B radiation acts as a significant environmental filter in arid ecosystems. In this study, the optimal UVB-2 range for *R. songarica* was identified as 165,841.44-199,758.31 J·m^-2^ day^-1^ (**Table 1**; **Figure 5**). The species’ adaptation to this narrow window is linked to its physiological capacity for flavonoid and anthocyanin synthesis, as well as UV-B tolerance (Liu et al., 2013). Liu et al. (2013) showed that *R. songarica* modulates the RsF3H gene to initiate the synthesis of flavonoids and anthocyanins. These compounds absorb UV-B radiation (280–320 nm) and mitigate reactive oxygen species (ROS), thereby protecting the photosynthetic organs. This optimal range enhances RsF3H expression; deviations, whether excessive or insufficient, hinder growth, while the accumulation of flavonoids improves tolerance to both UV-B and drought. This underscores the critical limiting role of UVB-2 in the distribution of *R. songarica*.

Precipitation is a critical variable influencing the establishment and survival of species (Lv et al., 2025). Optimal water and nutrient conditions are essential for seed germination and plant development (He et al., 2020). Although plants in arid and semi-arid regions exhibit strong drought tolerance, water remains a key limiting factor for their growth and distribution. This study indicates that the optimal annual precipitation for the growth of *R. songarica* ranges from 44.46 mm to 164.49 mm (**Table 1**). The finding aligns well with the arid and semi-arid climate conditions characteristic of the species’ distribution area. According to Wang P. et al. (2020), annual precipitation was shown to be an important environmental factor limiting the growth and distribution of *R. songarica*. This finding aligns with the results of this study, suggesting that the effects of precipitation on species establishment and survival, as well as the expansion or contraction of natural populations, are contingent upon future precipitation patterns.

Temperature plays a crucial role in the growth and development of plants, significantly influencing their geographical distribution (Wang and Shang, 2020). Research indicates that variations in the global distribution of species are largely determined by their cold tolerance levels (Araújo et al., 2013). Additionally, the temperature differential is among the most critical factors influencing plant growth, development, and flowering (Xie et al., 2022). Most plants require exposure to alternating warm and cold stages during these vital growth periods (Feng et al., 2025). The Mean Temperature of the Coldest Quarter (Bio11) suitable for the survival of *R. songarica* ranges from −15.91 °C to −3.62 °C, indicating a narrow suitable range and reflecting its strong cold hardiness (**Table 1**). The results indicate that, under current climatic conditions, *R. songarica* is primarily distributed in the core regions of Central Asia, including the Mongolian Plateau, Xinjiang, and western Inner Mongolia (**Figure 6**). The dry, cold climate characterized by significant temperature fluctuations between day and night, along with high ultraviolet radiation, fosters the growth of *R. songarica*, highlighting its preference for drought and low-temperature resistance. Additionally, in their investigation of the distribution patterns of endemic plant groups, Zhang et al. (2017) observed that many plants, including *R. songarica*, tend to thrive in arid climates. Our findings align with theirs.

The distribution of *R. songarica* is determined not by individual environmental factors, but by their interactions. UV-B stress is modulated by both temperature and moisture: cold winters exacerbate oxidative stress by suppressing flavonoid synthesis (Liu et al., 2013), while water availability regulates UV-B tolerance, with drought limiting flavonoid production (Wang et al., 2025) and precipitation supporting it (Wang A. et al., 2023). Additionally, Bio11 and Bio12 co-vary in high-altitude regions, where cold and drought stresses combine to select for enhanced tolerance (Wu et al., 2014; Yang Y. et al., 2025). In summary, these interlinked factors ultimately confine *R. songarica* to arid and semi-arid environments with low temperatures, limited rainfall, and high UV radiation.

### 4.3 Environmental impact of geographical distribution changes of *R. songarica* under different climate change scenarios

Climate change has altered the structure and function of terrestrial ecosystems, thereby impacting the habitat and geographic distribution of species (Vintsek et al., 2024). In this study, we employed the MaxEnt model to predict changes in the potential geographic distribution of *R. songarica* under current and future climate change scenarios, specifically for the 2030s, 2050s, 2070s, and 2090s, across SSP126, SSP245, SSP370, and SSP585 (**Figure 6** and **7**). The prediction results indicated that the total potential suitable distribution area for *R. songarica* during the current period is 46.44 × 10^6^ km². Of this, the extremely highly suitable area comprises 28.75% (13.35 × 10^6^ km²), primarily located in the core regions of Central Asia, including the Mongolian Plateau, Xinjiang, and western Inner Mongolia. The highly suitable area constitutes 16.13% (7.49 × 10^6^ km²), predominantly found in Central Asia, West Asia, and Northwest China. The moderately suitable area accounts for 20.28% (9.42 × 10^6^ km²) and is mainly situated in the central part of North America. The poorly suitable area represents 34.82% (16.17 × 10^6^ km²) and is dispersed across regions such as central North America, a small area of southern Africa, parts of West Asia, and the eastern edge of China. This distribution pattern indicates that the habitat of *R. songarica* is highly concentrated in the temperate arid and semi-arid regions of Eurasia, with its survival reliant on specific environmental conditions, including drought and a temperate continental climate. The model simulation results align with the species’ ecological adaptability characteristics, suggesting that the simulation was effective.

Aligning with the findings of Wang et al. (2024), under the four future scenarios, the potential suitable distribution areas for *R. songarica* exhibit a consistent trend of reduction, with varying degrees of shrinkage across different climate scenarios. This decline may be attributed to increased CO_2_ emissions, intensified human activities, and the frequent occurrence of extreme climate events, all of which alter the water and thermal conditions in the originally suitable areas, thereby diminishing habitat suitability. In the SSP126 scenario, the potential suitable distribution areas for *R. songarica* experienced only a slight reduction, showing no significant change from the present to the future (**Figures 6**, **7**). This finding aligns with the results of Wei et al. (2021) study on *Ophiocordyceps sinensis*. The stable contraction of suitable areas in the low-emission scenario may result from rising greenhouse gas concentrations, which modify the hydrothermal conditions in the potential growth areas for *R. songarica*, while the increasing frequency of extreme climate events exacerbates the reduction of these potential distribution areas. In the 2090-SSP245 scenario, the areas deemed potentially suitable for distribution will experience a significant reduction, particularly in poorly suitable regions, which will see a decrease of -15.89%. Conversely, the extremely highly suitable areas will remain largely stable. In the 2090-SSP370 scenario, however, the moderately suitable areas will contract sharply, with a reduction rate of -23.67%. Additionally, both the highly suitable and extremely highly suitable areas will continue to diminish, with reductions of -15.89% and -6.97%, respectively. This trend illustrates a phenomenon of core contraction and edge fragmentation, aligning with the findings of Shi et al. (2024) regarding *Quercus gilva*. Under future climatic conditions, the potential distribution areas for *R. songarica* are expected to shrink as climate anomalies increase, suggesting that climate change poses a significant threat to the survival of *R. songarica*. Research indicates that global warming negatively impacts populations, communities, and ecosystems, thereby influencing species distribution (Geng et al., 2022). As temperatures rise, plant species are likely to migrate to higher altitudes or latitudes (Brüssow et al., 2024; Lai et al., 2023; Ma et al., 2020). This study corroborates those findings, potentially attributing the observed changes to ongoing climate warming driven by increased greenhouse gas concentrations, which may create more favorable conditions for growth in plateau climate areas characterized by *R. songarica*. In the 2050-SSP585 scenario, the highly suitable habitat in the core region of Central Asia diminishes into an isolated patch, while the poorly suitable area in central North America essentially vanishes. These findings align with the results reported by Puchalka et al. (2023) Climate change is expected to facilitate the emergence of species in most regions that were previously suitable. The warming climate, driven by increased greenhouse gas concentrations and the frequent occurrence of extreme weather events, exacerbates the shrinkage and fragmentation of habitats (Lv et al., 2025). The reduction in both the total suitable area and the area of each suitability grade across various emission scenarios further underscores the significant impact of greenhouse gas emission intensity on changes in specie habitats.

The centroid migration of potential suitable distribution areas for *R. songarica* exhibits a distinct core trend: it migrates from west to east along the path: Caucasus→Central Asian arid area→Xinjiang and eastern arid area of China (**Figure 8**; **Table 4**). Notably, higher greenhouse gas emission intensities correlate with longer cumulative migration distances of the centroid. Differences in centroid migration paths emerge across various emission scenarios. Under low emission scenarios (SSP126 and SSP245), the centroid migration demonstrates a phased characteristic of ‘slow eastward movement-medium-term stability-followed by rapid transition.’ In contrast, under medium-high and high emission scenarios (SSP370 and SSP585), the centroid continues to advance toward inland arid areas, ultimately migrating to the Tarim Basin and the Qilian Mountains-Hexi Corridor in Xinjiang, China. All four scenarios indicate that the core suitable area for *R. songarica* will gradually shift from the western portion of the Eurasian arid zone to the central and eastern regions. This shift is attributed to the greater suitability of the eastern arid core area for its growth, while the western region is increasingly impacted by climate warming. Contrary to the findings of Wang et al. (2024), who predicted a shift towards higher latitudes within China, our global-scale analysis projects a longer eastward migration route. This discrepancy can be attributed to the differing spatial scales and the larger number of distribution points included in our model.

Habitat shrinkage and fragmentation of *R. songarica* are increasingly severe. Higher greenhouse gas emission intensities correlate with more pronounced habitat reduction and fragmentation. Climate change significantly impacts its habitat, threatening its survival, whether through global shrinkage and core area isolation under high emission scenarios or local stability with slight shrinkage in low emission scenarios. The migration of the centroid of the suitable area for *R. songarica* toward the eastern arid core area holds considerable importance for the regional desert ecosystem, vegetation restoration, and sand control strategies. In the future, it is essential to enhance the protection of *R. songarica* population in the eastern arid regions of Central Asia, specifically in Xinjiang and the Qilian Mountains, in alignment with the centroid migration trend to mitigate the adverse effects of climate change.

### 4.4 Conservation strategies and recommendations

Studying the response of species’ geographical distributions to climate change is crucial for providing a scientific basis for species protection (Zhang and Wang, 2023; Dong et al., 2022). Some researchers have identified that areas suitable for species distribution in the context of climate change often serve as “climatic havens” for species survival (Denney et al., 2020). Our research indicates that, under both current and future scenarios, the most suitable habitat for the growth of *R. songarica* is situated in the core region of Central Asia, encompassing the Mongolian Plateau, Xinjiang, and western Inner Mongolia. Furthermore, projections across four future scenarios suggest that the core suitable area will gradually shift from the western part of the Eurasian arid zone to the eastern arid regions of Central Asia, specifically the Xinjiang-Qilian Mountains in the central and eastern sections (**Figure 9**). Establishing reserves or germplasm resources for *R. songarica* in these areas can effectively safeguard the genetic diversity of this species. Furthermore, the findings indicate that the low to medium suitability regions in central North America are projected to diminish significantly or vanish entirely under future high-emission scenarios. The forecasts suggest a continued decline in the potential suitable habitats of *R. songarica*, with a more pronounced decrease in areas classified as medium to low suitability. The central habitat is susceptible to fragmentation in high-emission scenarios, while stability in suitable habitats is only evident in low-emission scenarios. These alterations stem from climate warming induced by rising greenhouse gas levels and the escalating occurrence of extreme climatic events, which alter the hydrothermal conditions of the original suitable habitats, diminishing habitat suitability. Whether it is the decline of potential habitats in high-concentration emission scenarios or the marginal retraction in low-concentration emission scenarios, climate change significantly impacts *R. songarica* habitats. The species faces a severe threat to its survival due to climate change, necessitating increased protection measures to safeguard *R. songarica* from future climatic impacts.

## 5 Conclusion

This study investigated the impacts of climate change on the potential distribution of *R. songarica* using species distribution modeling. The key factors limiting its distribution were UV−B seasonality, mean temperature of the coldest quarter, and annual precipitation, with UV−B seasonality being the most critical. At present, the climatically suitable habitats of *R. songarica* are mainly located in the temperate arid and semi−arid regions of Eurasia, with Central Asia as the core distribution area. Under future climate scenarios, the total suitable area is predicted to shrink gradually, and the reduction will become more pronounced under high−emission scenarios. The reduced habitats are mainly distributed around the current core region, and the centroid of the suitable range is projected to shift eastward toward Central Asia, Xinjiang, and the Qilian Mountains. These results provide a scientific basis for the conservation, cultivation, and management of *R. songarica* and its desert ecosystems.

## Data Availability

The original contributions presented in the study are included in the article/supplementary material. Further inquiries can be directed to the corresponding author.
